# Fish Oil Supplementation Reduces Heart Levels of Interleukin-6 in Rats with Chronic Inflammation due to Epilepsy

**DOI:** 10.3389/fneur.2017.00263

**Published:** 2017-06-09

**Authors:** Mariana Bocca Nejm, André Abou Haidar, Aparecida Emiko Hirata, Lila Missae Oyama, Antonio-Carlos Guimarães de Almeida, Roberta Monterazzo Cysneiros, Esper Abrão Cavalheiro, Carla Alessandra Scorza, Fulvio Alexandre Scorza

**Affiliations:** ^1^Disciplina de Neurociência, Escola Paulista de Medicina/Universidade Federal de São Paulo (EPM/UNIFESP), São Paulo, Brazil; ^2^Departamento de Fisiologia, Escola Paulista de Medicina/Universidade Federal de São Paulo (EPM/UNIFESP), São Paulo, Brazil; ^3^Departamento de Engenharia de Biossistemas, Universidade Federal de São João Del Rei (UFSJ), São João Del Rei, Brazil; ^4^Programa de Pós-Graduação em Distúrbios do Desenvolvimento do Centro de Ciências Biológicas e da Saúde da Universidade Presbiteriana Mackenzie, São Paulo, Brazil

**Keywords:** epilepsy, sudden unexpected death in epilepsy, inflammation, heart, interleukin-6, fish oil

## Abstract

Sudden unexpected death in epilepsy (SUDEP) is a major cause of premature death related to epilepsy. The causes of SUDEP remain unknown, but cardiac arrhythmias and asphyxia have been suggested as a major mechanism of this event. Inflammation has been implicated in the pathogenesis of both epilepsy and ventricular arrhythmia, with interleukin-6 (IL-6) being recognized as a crucial orchestrator of inflammatory states. Our group previously reported that levels of IL-6 were increased in the hearts of epileptic rats. In this scenario, anti-inflammatory actions are among the beneficial effects of fish oil dietary supplementation. This investigation revealed that elevated levels of IL-6 in the heart were markedly reduced in epileptic rats that were treated in the long-term with fish oil, suggesting protective anti-inflammatory actions against dangerously high levels of IL-6. Based on these findings, our results suggest beneficial effects of long-term intake of fish oil in reducing the inflammation associated with chronic epilepsy.

## Introduction

The mortality rate in people with epilepsy is substantially higher than that observed in the general population, which is a matter of concern among specialists (1). In this scenario, sudden unexpected death in epilepsy (SUDEP) is a leading cause of premature mortality directly related to epilepsy (2).

Sudden unexpected death in epilepsy is defined as “sudden, unexpected, witnessed or unwitnessed, non-traumatic and non-drowning death in patients with epilepsy, with or without evidence for a seizure, and excludes documented *Status epilepticus* (SE), in which postmortem examination does not reveal a toxicological or anatomic cause for death” (3). The fatal event is most likely provoked by functional disturbance, and researchers have proposed cardiac arrhythmia as a potential cause of SUDEP (2). Recently, clinically relevant genetic variants in cardiac arrhythmia and epilepsy have been found in a considerable number of SUDEP cases (4). Inflammation has been implicated in the pathophysiological events of both epilepsy and life-threatening ventricular arrhythmia (5, 6), in which the central role of the interleukin-6 (IL-6) is well recognized.

Our group was the first to describe increased levels of IL-6 in the hearts of rats with chronic epilepsy (7). Omega-3 polyunsaturated fatty acids, which are abundant in fish oil, have been described as potent anti-inflammatory agents (8). Thus, in the search for complementary therapy for epilepsy, this study was conducted to examine the potential anti-inflammatory effects of fish oil supplementation on the levels of the pro-inflammatory cytokine IL-6 in the heart of rats with chronic epilepsy.

## Animals and Methods

### Animals

Adult male Wistar rats (220–280 g) were housed under standard controlled conditions (7:00 a.m./7:00 p.m. light/dark cycle; 20–22°C; 45–55% humidity) with food and water *ad libitum*. All animal experiments were carried out in accordance with the National Institutes of Health guide for care and use of laboratory animals and approved by the Animal Care Committee of UNIFESP (CEUA 188439).

### Induction of Epilepsy

Epilepsy was induced according to the procedure described previously (9). In brief, 30 min after methyl scopolamine injection (1 mg/kg, s.c.—Sigma, MO, USA), pilocarpine was administered (350 mg/kg, i.p.—Sigma, MO, USA). Animals developed SE. To terminate SE, diazepam (10 mg/kg—Cristalia, Compaz) was administered subcutaneously 3 h after SE onset. Rats evolved through the latent period to the chronic phase of the pilocarpine model. To confirm the presence of epilepsy, animals were continuously video monitored for 60 days, and spontaneous recurrent seizures observed. At the end, nine rats with epilepsy (*n* = 9) were used in the experiments.

### Fish Oil Treatment

Animals were randomly divided into the following groups: (1) animals treated daily with vehicle (0.009% cremophor) (control vehicle); (2) animals treated daily with 85 mg/kg fish oil (control fish oil); (3) animals with epilepsy treated daily with vehicle (epilepsy vehicle); and (4) animals with epilepsy treated with 85 mg/kg fish oil (epilepsy fish oil). During the 90-day treatment, animals received vehicle (cremophor 0.009%) or fish oil (PROEPA^®^, 85 mg/kg). A single daily dose was given *via* oral gavage between 11:00 and 12:00 a.m. Volume was adjusted according to animal weight, which was verified three times a week during the 90-day treatment period. Each fish oil capsule contained eicosapentaenoic acid (EPA), 180 mg, and docosahexaenoic acid (DHA), 120 mg. Capsule contents were dissolved in 0.009% cremophor, yielding a final concentration of 21.25 mg/ml fish oil, corresponding to 3.82 mg/ml EPA and 2.55 mg/ml DHA, and the final composition of fish oil was administered as 1 ml/250 g of body weight. Animals were killed by decapitation. Hearts were removed, and ventricles were separated and split in half (for Western blot and ELISA procedures) and then stored at −80°C until use.

### Measurements of IL-6 Protein Levels

One half of the ventricle was used for the Western blotting procedure, and the other half was used for the ELISA assay. For Western blot analysis, samples were prepared as previously described by Nejm et al. (10). The primary antibodies used were anti-IL-6 (Millipore-Chemicon, USA) and anti-β-actin (Sigma Aldrich, USA). ELISA assays (DuoSet ELISA, R&D Systems, Minneapolis, MN, USA) were performed following the recommendations of the manufacturer. All samples were run in duplicate, and the mean value was reported.

### Statistical Analysis

Data were expressed as the mean ± standard error. Statistical analysis was performed using two-way ANOVA [grouped as control versus experimental and by treatment (vehicle versus fish oil)] followed by Bonferroni posttest. *p* Values of 0.05 or less were considered significant.

## Results

**Figure 1:**
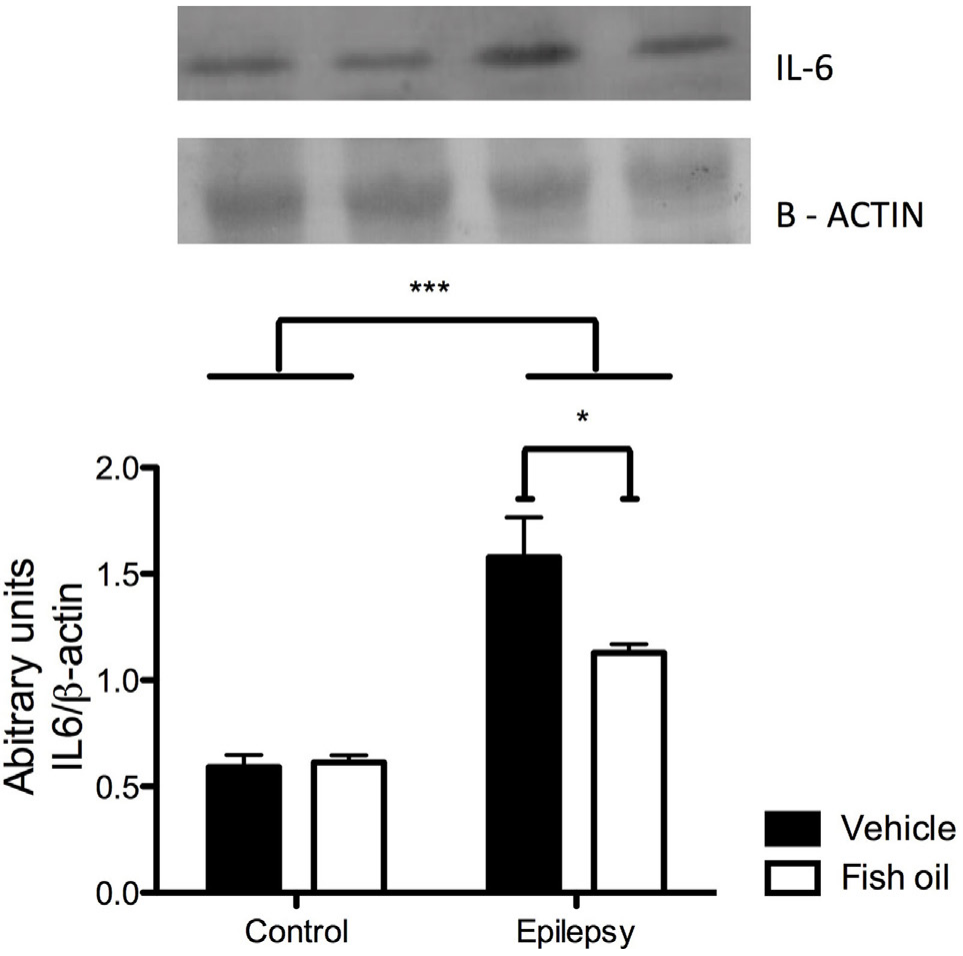
Immunoblot of interleukin-6 (IL-6). IL-6 levels in hearts of rats from control and epilepsy groups treated with vehicle or fish oil (85 mg/kg). The PVDF membrane was probed with anti-IL-6 antibody and re-probed with anti-β-actin. Each bar represents the mean ± SEM of the ratio IL-6/β-actin from four individual experiments (*p < 0.05; ***p < 0.001).

The results of Western blot analysis showed that IL-6 levels in the heart of rats with epilepsy were substantially increased when compared with control rats [*F*(1;12) = 54.78; *p* < 0.0001]. Animals with epilepsy that were treated with fish oil for an extended time exhibited a marked reduction in IL-6 levels, since an interaction effect was observed between epilepsy and fish oil treatment [*F*(1;12) = 5.34; *p* = 0.039], as shown in Figure 1.

**Figure 2:**
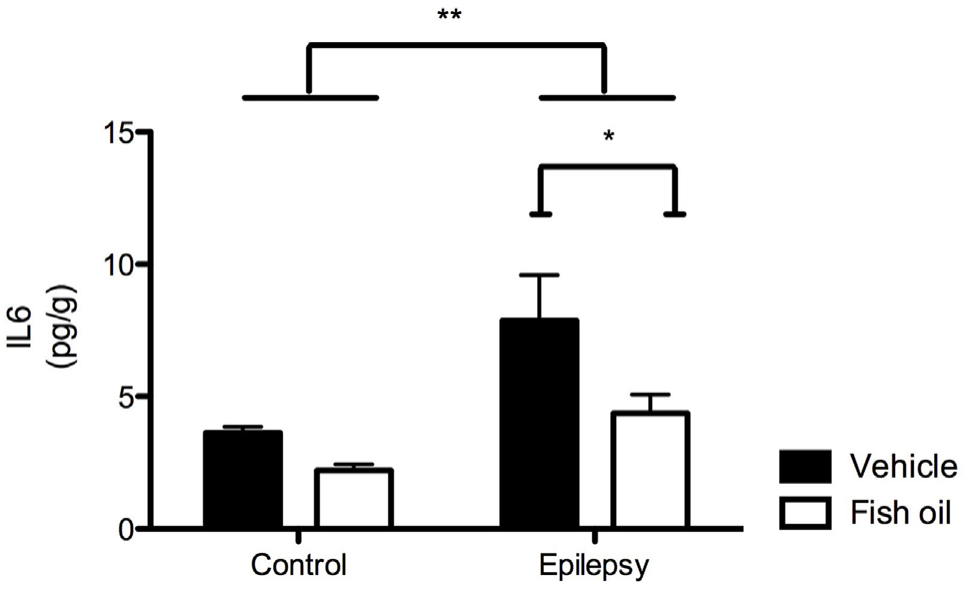
Interleukin-6 (IL-6) quantification by ELISA. IL-6 levels in the hearts of rats in control and epilepsy groups treated with vehicle or fish oil (85 mg/kg) for 90 days. Each bar represents mean ± SEM of five individual experiments (*p < 0.05; **p < 0.01).

In Figure 2, the ELISA results corroborated the results obtained with immunoblotting analysis as increased levels of IL-6 were found in the hearts of animals with epilepsy compared with those in control rats [*F*(1;16) = 10.26; *p* = 0.0059]. Furthermore, the ELISA results show the effectiveness of the fish oil treatment, which reduced IL-6 levels in both control and epileptic rats [*F*(1;16) = 6.05; *p* = 0.0264]. There was no interaction effect between epilepsy and fish oil treatment [*F*(1;16) = 1.06; *p* = 0.3178].

## Discussion

In this rodent pilocarpine model of seizures, fish oil supplementation reduced cardiac levels of IL-6, indicating an anti-inflammatory effect of fish oil.

Consistent preclinical and clinical findings support the presence of a persistent inflammatory condition in the brain and plasma of subjects with epilepsy (4, 6, 11). Epileptic seizures often affect heart rate and rhythm, and poor seizure control is by far the major clinical risk factor for SUDEP (2). However, despite the available therapeutic arsenal, 30–40% of individuals with epilepsy have uncontrolled seizures. Studies focused on the pathological basis of epilepsy have focused on the role of inflammatory events (6). Basic and clinical studies has been described that chronic inflammatory state contribute to the pathogenesis of seizures and maintance of epilepsy (6, 11, 12). Previously, we demonstrated chronically elevated levels of the pro-inflammatory cytokine IL-6 in the ventricles of rats with seizures (7). Here, our findings support the existence of persistent inflammation in the ventricles of rats with epilepsy, which exhibited elevated levels of IL-6. Elevated circulating levels of IL-6 have been described in patients with heart failure, and these higher levels are positively correlated with the presence and duration of cardiac arrhythmias and with the severity of disease and mortality risk (5, 13). Taken together, there is compelling evidence that increased levels of IL-6 are deeply involved in the pathophysiological mechanisms underlying both epilepsy and cardiac diseases.

Using the pilocarpine model of epilepsy, Ferrari and colleagues in our group demonstrated that chronic treatment with fish oil promoted neuroprotection and positive plastic changes in the brain of rats with epilepsy (14). The beneficial anti-inflammatory effects of omega-3 fatty acids in chronic inflammatory diseases have been consistently documented (8). Fish oil dietary consumption is suggested as a good source of omega-3 polyunsaturated fatty acids for consumers. This study suggests that chronic oil fish supplementation has a protective anti-inflammatory effect against elevated levels of IL-6 in the heart of rats with epilepsy, but the underlying mechanisms of these effects remain unknown.

## Conclusion

Our findings report the beneficial anti-inflammatory effects of long-term oil fish intake in the hearts of rats with chronic inflammation associated with epilepsy. Our results suggest the potential therapeutic value of dietary supplementation with oil fish in patients with epilepsy.

## Ethics Statement

All animals were treated according to protocols for animal care, and this study was carried out in accordance with the recommendations established by the Federal University of Sao Paulo. All efforts were made to minimize animal suffering (Comissão de Ética no Uso de Animais—CEUA).

## Author Contributions

MN: conducted the experiments, acquired data, analyzed data, critically discussed the manuscript, and wrote the manuscript. AAH: conducted the experiments, acquired data, analyzed data, and critically discussed the manuscript. AEH, LO, A-CA, and RC: analyzed data and critically discussed the manuscript. EC: conceived the work and wrote the manuscript. CS: critically discussed the manuscript and wrote the manuscript. FS: conceived the work, critically discussed the manuscript, and wrote the manuscript.

## Conflict of Interest Statement

The authors declare that the research was conducted in the absence of any commercial or financial relationships that could be construed as a potential conflict of interest.

## References

[1] Gaitatzis A Johnson AL Chadwick DW Shorvon SD Sander JW. Life expectancy in people with newly diagnosed epilepsy. Brain (2004) 127:2427–32. 10.1093/brain/awh267 15371287

[2] Surges R Thijs RD Tan HL Sander JW Sudden unexpected death in epilepsy: risk factors and potential pathomechanisms. Nat Rev Neurol (2009) 5:492–504. 10.1038/nrneurol.2009.118 19668244

[3] Nashef L. Sudden unexpected death in epilepsy: terminology and definitions. Epilepsia (1997) 38:S6–8. 10.1111/j.1528-1157.1997.tb06130.x 19909329

[4] Bagnall RD Crompton DE Petrovski S Lam L Cutmore C Garry SI Exome-based analysis of cardiac arrhythmia, respiratory control, and epilepsy genes in sudden unexpected death in epilepsy. Ann Neurol (2016) 79:522–34. 10.1002/ana.24596 26704558

[5] Bozkurt B Mann DL Deswal A. Biomarkers of inflammation in heart failure. Heart Fail Rev (2010) 15:331–41. 10.1007/s10741-009-9140-3 19363700

[6] Vezzani A Epilepsy and inflammation in the brain: overview and pathophysiology. Epilepsy Curr (2014) 14:3–7. 10.5698/1535-7511-14.s2.3 24955068 PMC3966641

[7] Nejm MB Haidar AA Hirata AE Arida RM Naffah-Mazacoratti M da G Interleukin-6 bares a dark side in sudden unexpected death in epilepsy. Epilepsy Behav (2012) 24:285–6. 10.1016/j.yebeh.2012.04.113 22561097

[8] Calder PC. Fatty acids and inflammation: the cutting edge between food and pharma. Eur J Pharmacol (2011) 668:S50–8. 10.1016/j.ejphar.2011.05.085 21816146

[9] Cavalheiro EA. The pilocarpine model of epilepsy. Ital J Neurol Sci (1995) 16:33–7. 10.1007/BF02229072 7642349

[10] Nejm MB Haidar AA Marques MJ Hirata AE Nogueira FN Cavalheiro EA Fish oil provides protection against the oxidative stress in pilocarpine model of epilepsy. Metab Brain Dis (2015) 30:903–9. 10.1007/s11011-015-9666-0 25893881

[11] Uludag IF Duksal T Tiftikcioglu BI Zorlu Y Ozkaya F Kirkali G IL-1β, IL-6 and IL1Ra levels in temporal lobe epilepsy. Seizure (2015) 26:22–5. 10.1016/j.seizure.2015.01.009 25799897

[12] Varella PP Santiago JF Carrete H Jr Higa EM Yacubian EM Centeno RS Relationship between fluid-attenuated inversion-recovery (FLAIR) signal intensity and inflammatory mediator’s levels in the hippocampus of patients with temporal lobe epilepsy and mesial temporal sclerosis. Arq Neuropsiquiatr (2011) 69:91–9. 10.1590/S0004-282X2011000100018 21359430

[13] Swerdlow DI Holmes MV Kuchenbaecker KB Engmann JE Shah T Sofat R The interleukin-6 receptor as a target for prevention of coronary heart disease: a Mendelian randomisation analysis. Lancet (2012) 379:1214–24. 10.1016/S0140-6736(12)60110-X 22421340 PMC3316968

[14] Ferrari D Cysneiros RM Scorza CA Arida RM Cavalheiro EA de Almeida AC Neuroprotective activity of omega-3 fatty acids against epilepsy-induced hippocampal damage: quantification with immunohistochemical for calcium-binding proteins. Epilepsy Behav (2008) 13:36–42. 10.1016/j.yebeh.2008.01.001 18295545

